# Metachronous sequential involvement of rice-body bursitis in the shoulder and knee: a case report of rare polyarticular presentation in a patient with rheumatoid arthritis

**DOI:** 10.3389/fmed.2026.1977597

**Published:** 2026-10-21

**Authors:** HanYing Mei, SenGuo Ji, Xuming Zhang, Xiaoping Shen

**Affiliations:** 1 Department of Rheumatology and Immunology, Jiujiang City Key Laboratory of Cell Therapy, Jiujiang NO.1 People’s Hospital Jiujiang, Jiangxi, China; 2 Department of Neurology, Jiujiang City Key Laboratory of Cell Therapy, Jiujiang NO.1 People’s Hospital Jiujiang, Jiangxi, China

**Keywords:** arthroscopic synovectomy, knee, metachronous involvement, rheumatoid arthritis, rice-body bursitis, shoulder

## Abstract

Rice-body bursitis is an uncommon complication of rheumatoid arthritis (RA). Metachronous involvement of multiple joints has rarely been described. We report a 52-year-old woman with a 16-year history of RA who presented in 2019 with refractory swelling of the right shoulder. Ultrasound and MRI revealed “honeycomb-like” effusion and T2-hypointense particles within the subacromial-subdeltoid bursa, leading to a probable imaging-based diagnosis of rice-body bursitis. She achieved complete remission approximately 6 months after adalimumab was started, during which leflunomide was continued; adalimumab was then discontinued by the patient. In April 2026, left knee swelling recurred, ESR was 37 mm/h and CRP was 1.842 mg/dl, while other joint symptoms were minimal. MRI showed numerous floating particles and T2-low-signal bodies, and arthroscopic exploration confirmed rice-body bursitis of the knee. After arthroscopic debridement, the knee symptoms resolved completely within one week. The two episodes occurred 7 years apart and involved different joints, indicating that rice-body bursitis in RA can present as a metachronous sequential condition. Inflammatory marker levels did not parallel the severity of local joint involvement in this patient. This case underscores that rice-body bursitis should be considered in RA patients with refractory monoarthritis. Arthroscopic clearance combined with systemic antirheumatic therapy was followed by resolution of knee symptoms within one week in this patient. Because the two episodes involved different joints, disease phases, and concurrent treatments, no comparison between treatment strategies can be made.

## Introduction

Rheumatoid arthritis（RA) can be complicated by rice-body bursitis, a rare condition characterized by the formation of numerous smooth, rice-like fibrinoid bodies within bursae (1–4). This phenomenon can also occur in systemic lupus erythematosus (1). The pathogenesis is thought to involve chronic synovial inflammation with deposition of fibrin, collagen, and immune complexes (5, 6). The shoulder and knee are the most frequent sites, but other joints such as the wrist can also be involved (7, 8). The clinical presentation—local swelling, pain, and limited motion—is non-specific and often mistaken for active RA or septic arthritis, leading to diagnostic delays (3, 4). Ultrasound and magnetic resonance imaging (MRI) play a pivotal role; typical findings include “honeycomb” effusion and T2-hypointense floating particles (9).

To date, published cases of RA-associated rice-body bursitis have almost exclusively involved a single joint (2–6); metachronous involvement of multiple joints in the same patient has not been documented. Here we describe a 52-year-old woman with RA who developed right-shoulder rice-body bursitis in 2019, which resolved after adalimumab therapy, and then presented 7 years later with left-knee rice-body bursitis confirmed by MRI and arthroscopy. The occurrence of a new rice-body lesion despite sequential treatment with a TNF-α inhibitor and a JAK inhibitor suggests that systemic anti-inflammatory therapy may not fully prevent the formation of these bodies. This report emphasizes that when RA patients present with refractory, asymmetrical monoarticular swelling, rice-body bursitis should be suspected and appropriate imaging obtained. For sequential involvement, a combination of systemic disease-modifying therapy and arthroscopic debridement may be considered; however, whether this approach is superior to pharmacotherapy alone cannot be determined from this single case.

## Case presentation

Patient Information and Timeline 2003, Diagnosed with rheumatoid arthritis; started methotrexate and leflunomide 2009–2010, Received methotrexate plus etanercept for 6 months with good response May 2019, Right shoulder swelling developed and progressively worsened July 2019, Admitted; probable imaging-based diagnosis of right-shoulder rice-body bursitis; started adalimumab 40 mg every 2 weeks October 2019–March 2020, Adalimumab reduced to monthly injections April 2020, Discontinued adalimumab on her own; continued leflunomide and etoricoxib May 2023, Added tofacitinib citrate for recurrent hand joint symptoms November 2025 Left knee pain and swelling; improved after local betamethasone injection April 2026，Left knee swelling recurred; MRI confirmed rice-body bursitis; Arthroscopic synovectomy performed;Adalimumab was restarted postoperatively at 40 mg every other week, together with leflunomide 20 mg daily September 2026 (5months post-op)，Knee symptoms resolved within one week; maintained on adalimumab plus leflunomide.

A 52-year-old woman was admitted in July 2019 with a 2-month history of progressive right-shoulder swelling. She had a 16-year history of Rheumatoid arthritis（RA) at that time, initially diagnosed in 2003 when she developed symmetrical polyarthritis involving the proximal interphalangeal, wrist, metacarpophalangeal, and knee joints, with morning stiffness. She had previously been treated with methotrexate (10 mg weekly), leflunomide (20 mg nightly), and non-steroidal anti-inflammatory drugs (meloxicam, loxoprofen), which provided partial control. From 2009 to early 2010, she received methotrexate (12.5 mg weekly) plus etanercept (25 mg weekly) for 6 months, with good response. Thereafter, she continued methotrexate (12.5 mg weekly), leflunomide (10 mg nightly), and aceclofenac (0.1 g twice daily), with acceptable disease control.

In May 2019, right-shoulder swelling developed and worsened progressively, accompanied by bilateral ankle swelling and pain. Her past medical history was unremarkable. She had no significant family history of autoimmune or rheumatic diseases. Her reproductive history was unremarkable.

### Clinical findings

On admission, vital signs were stable. Physical examination revealed marked right-shoulder enlargement without significant tenderness, and bilateral ankle swelling with tenderness but preserved range of motion. The rotator cuff showed intact but mildly restricted active abduction, with no obvious tear on clinical testing. Glenohumeral joint motion was limited in forward flexion and external rotation due to pain and swelling.

Laboratory results: rheumatoid factor 20.9 IU/ml（reference range: 0–20 IU/ml, slightly elevated), anti-cyclic citrullinated peptide antibody 68.23 RU/ml（reference range: 0–17 RU/ml, elevated), C-reactive protein 0.884 mg/dl（reference range: 0–0.5 mg/dl, slightly elevated), erythrocyte sedimentation rate 18 mm/h（reference range: 0–20 mm/h, within normal limits), immunoglobulin G 1630.0 mg/dl（reference range: 700–1600 mg/dl, slightly elevated). Anti-perinuclear factor and anti-filaggrin antibodies were negative（normol), as was antinuclear antibody (1:100). Tumour markers were normal.

Shoulder ultrasound (June 2019) showed effusion in the subacromial-subdeltoid bursa with poor acoustic transparency and a honeycomb pattern. MRI (3.0-T scanner, T1-weighted and T2-weighted sequences with fat suppression, slice thickness 3 mm) revealed cortical irregularity and multiple diffuse nodular lesions in the periarticular soft tissues. These lesions appeared T1-hypointense and T2-hyperintense, reflecting the surrounding inflammatory soft-tissue reaction; the T2-hypointense particles described in the literature as a diagnostic hallmark of rice-body bursitis correspond to the synthetic particle burden itself and were identified separately on dedicated T2-weighted sequences. Enlarged lymph nodes were also noted in the right supraclavicular and axillary regions. The glenohumeral joint space was preserved without significant cartilage erosion.

### Diagnostic assessment

Based on the clinical, laboratory, and imaging features, a diagnosis of RA with right-shoulder rice-body bursitis was established. The shoulder lesion was diagnosed as a probable imaging-based diagnosis, as the patient declined ultrasound-guided aspiration or biopsy and no histological or microbiological confirmation was obtained. No microbiological examination was performed for the shoulder at that time. The patient had no fever, night sweats, or other systemic signs suggestive of infection, and empirical antibiotic therapy was not administered. This prolonged course without anti-infective treatment supports, but does not confirm, a non-infectious aetiology and does not constitute microbiological exclusion. The differential diagnosis included infectious bursitis (particularly tuberculous or atypical mycobacterial), gout, and calcium pyrophosphate deposition disease. However, the imaging features—particularly the honeycomb effusion and T2-hypointense intra-bursal particles—were considered characteristic of rice-body bursitis, and the clinical context of long-standing RA further supported this diagnosis.

### Therapeutic intervention

In July 2019, treatment was switched to adalimumab (Humira) 40 mg subcutaneously every 2 weeks, later reduced to monthly injections from October 2019 to March 2020, combined with leflunomide 20 mg daily and celecoxib 0.2 g daily. The shoulder swelling improved substantially. In April 2020 the patient discontinued adalimumab on her own, continuing leflunomide and etoricoxib. Adalimumab was therefore not administered between April 2020 and the knee surgery in April 2026. In May 2023, because of recurrent pain and swelling in the second and third proximal interphalangeal joints of both hands, tofacitinib citrate (one tablet twice daily) was added to leflunomide and etoricoxib. In November 2025, she developed left-knee pain and swelling; ultrasound revealed suprapatellar bursal effusion with synovial hyperplasia, which improved temporarily after local betamethasone injection. In April 2026, left-knee swelling recurred frequently, C-reactive protein 1.842 mg/dl（reference range: 0–0.5 mg/dl, slightly elevated), erythrocyte sedimentation rate 37 mm/h（reference range: 0–20 mm/h, elevated), MRI showed multiple floating echogenic particles and T2-low-signal bodies. Tofacitinib was discontinued because she developed herpes zoster. Arthroscopic synovectomy was performed, and a large number of rice bodies were removed. Adalimumab was restarted postoperatively at 40 mg every other week, together with leflunomide 20 mg daily then.

### Outcome, follow-up, and patient perspective

One week after surgery, the knee symptoms resolved completely. Maintenance therapy with adalimumab 40 mg every other week plus leflunomide 20 mg daily was continued. The patient reported satisfaction with the relief of knee symptoms after surgery. She also recalled that the shoulder episode had taken longer to improve; however, this recollection does not permit comparison between treatments. At the most recent follow-up, five months postoperatively, the knee remained stable with no recurrence of pain or swelling. The patient tolerated the maintenance regimen without significant adverse events.

### Pathological findings (knee episode)

Histological examination of the rice bodies and synovial tissue obtained during arthroscopic debridement revealed eosinophilic fibrinous material with chronic synovitis, consistent with rice-body formation. No granulomatous inflammation, caseous necrosis, or malignant cells were identified. Microbiological cultures (including for aerobic, anaerobic, and acid-fast organisms) were negative.

## Discussion

Although rice-body bursitis is a rare complication of RA, it should be included in the differential diagnosis when a patient with RA presents with refractory monoarticular swelling that responds poorly to conventional antirheumatic regimens (12, 15). There have even been reports of RA patients presenting with rice-body bursitis as the initial manifestation (10, 11). In our patient, both episodes featured prominent local joint swelling with minimal systemic polyarticular symptoms, underscoring that such “isolated” bursitis should prompt consideration of rice-body formation.

A unique aspect of this case is the metachronous sequential pattern: the right shoulder was affected in 2019 and the left knee in 2026, with a 7-year interval. Despite overall satisfactory disease control with adalimumab and subsequently tofacitinib, rice-body lesions emerged sequentially (12–14). This suggests that rice-body bursitis can involve multiple joints over time, and its occurrence may not be strictly synchronous with global RA activity. In the knee episode, ESR and CRP were elevated （ESR was 37 mm/h and CRP was 1.842 mg/dl), whereas the shoulder episode showed a normal ESR (18 mm/h) and only mildly elevated CRP (0.884 mg/dl). No formal composite disease activity score (DAS28, CDAI, or SDAI) was available at either episode, so we cannot draw conclusions regarding the relationship between rice-body formation and overall RA activity. The local inflammatory findings were not accompanied by a proportional systemic marker response, which may mislead clinicians toward ordinary synovitis or infection.

In terms of treatment outcome, the shoulder episode resolved approximately 6 months after adalimumab was started, and the knee symptoms resolved within one week after arthroscopic debridement combined with systemic antirheumatic therapy. These recovery times are reported descriptively only. Because the two episodes occurred in different joints, at different disease phases, and under different concurrent medications, no comparison between treatment strategies can be made, and no inference regarding the relative efficacy of surgery versus pharmacotherapy is warranted. A recent report by Xie et al. described arthroscopic management of shoulder rice-body bursitis in a patient with RA and discussed its mechanistic implications (2). Our case complements this report by demonstrating that rice-body formation can occur metachronously even with systemic biologic therapy, and that arthroscopic debridement was also performed in the knee, with resolution of symptoms (4). The diagnosis of rice-body bursitis relies heavily on imaging, particularly ultrasound and MRI. The characteristic honeycomb appearance on ultrasound and T2-hypointense intra-bursal particles on MRI carry high discriminatory value against simple synovitis or haemorrhagic effusion. Familiarity with these imaging hallmarks is important for early recognition. In our case, the shoulder lesion was diagnosed on imaging alone because the patient declined invasive confirmation. This remains a limitation, although the clinical course and follow-up findings are consistent with the diagnosis.

Several limitations should be acknowledged. First, this is a single-centre retrospective case report. Second, histological and microbiological confirmation were available only for the knee episode; the shoulder diagnosis was a probable imaging-based diagnosis, as the patient declined any invasive procedure at that time. Microbiological exclusion for the shoulder was therefore not performed. The long-term clinical course without anti-infective therapy argues against, but does not exclude, an infectious aetiology. Third, the long-term follow-up did not include serial imaging or laboratory monitoring, making it difficult to precisely define the temporal relationship between drug adjustments and rice-body development. Fourth, the patient's decision to discontinue adalimumab in 2019 introduced a confounding variable in interpreting the subsequent knee episode.

Future multicentre prospective studies with more RA-associated rice-body cases are needed to identify independent risk factors (such as disease duration, joint biomechanical load, local microenvironmental changes) and to refine individualized therapeutic strategies, including comparisons of different biologic agents and post-operative prophylactic regimens. Despite these limitations, this report's unique metachronous sequential presentation and the contrasting therapeutic responses across episodes offer valuable clinical insights for managing this rare complication.

## Data Availability

The original contributions presented in the study are included in the article/supplementary material, further inquiries can be directed to the corresponding author.
